# Somatostatin triggers rhythmic electrical firing in hypothalamic GHRH neurons

**DOI:** 10.1038/srep24394

**Published:** 2016-04-13

**Authors:** Guillaume Osterstock, Violeta Mitutsova, Alexander Barre, Manon Granier, Pierre Fontanaud, Marine Chazalon, Danielle Carmignac, Iain C. A. F. Robinson, Malcolm J. Low, Nikolaus Plesnila, David J. Hodson, Patrice Mollard, Pierre-François Méry

**Affiliations:** 1 Inserm U-1191, Montpellier, France; 2 CNRS UMR 5203, Institut de Génomique Fonctionnelle, Montpellier, France; 3 Université Montpellier, Montpellier, France; 4 Division of Molecular Neuroendocrinology, MRC National Institute for Medical Research, The Ridgeway, Mill Hill, London, UK; 5 Department of Molecular and Integrative Physiology, University of Michigan Medical School, Ann Arbor, MI, USA; 6 Royal College of Surgeons in Ireland, Dublin 2, Ireland; 7 Section of Cell Biology and Functional Genomics, Department of Medicine, Imperial College London, Imperial Centre for Translational and Experimental Medicine, Hammersmith Hospital, Du Cane Road, London W12 0NN, United Kingdom; 8 Institute of Metabolism and Systems Research (IMSR), University of Birmingham, Birmingham B15 2TT, United Kingdom; 9 Centre for Endocrinology, Diabetes and Metabolism, Birmingham Health Partners, Birmingham, B15 2TH, United Kingdom

## Abstract

Hypothalamic growth hormone-releasing hormone (GHRH) neurons orchestrate body growth/maturation and have been implicated in feeding responses and ageing. However, the electrical patterns that dictate GHRH neuron functions have remained elusive. Since the inhibitory neuropeptide somatostatin (SST) is considered to be a primary oscillator of the GH axis, we examined its acute effects on GHRH neurons in brain slices from male and female GHRH-GFP mice. At the cellular level, SST irregularly suppressed GHRH neuron electrical activity, leading to slow oscillations at the population level. This resulted from an initial inhibitory action at the GHRH neuron level via K^+^ channel activation, followed by a delayed, sst1/sst2 receptor-dependent unbalancing of glutamatergic and GABAergic synaptic inputs. The oscillation patterns induced by SST were sexually dimorphic, and could be explained by differential actions of SST on both GABAergic and glutamatergic currents. Thus, a tripartite neuronal circuit involving a fast hyperpolarization and a dual regulation of synaptic inputs appeared sufficient in pacing the activity of the GHRH neuronal population. These “feed-forward loops” may represent basic building blocks involved in the regulation of GHRH release and its downstream sexual specific functions.

Hypothalamic growth hormone-releasing hormone (GHRH) neurons control the pulsatile secretion of growth hormone (GH) from the pituitary gland^1^, thereby regulating growth and metabolism. In addition, these neurons are involved in the central regulation of glucose homeostasis^2^. However, the hypothalamic circuitry that allows GHRH neurons to deliver the appropriate spiking pattern in response to stimulation remains poorly characterized. Indeed, the underlying mechanisms may involve quantitative and qualitative changes at both the cell and population levels.

The small contingent of GHRH neurons (<2000) located in the arcuate nucleus project to the median eminence, where GHRH secretion into the adjacent portal system triggers pituitary GH release. Symmetrically, neuroendocrine somatostatin (SST) neurons concentrated within the periventricular nucleus also project to the median eminence where they release SST to exert an inhibitory control over GH release. Successful models of the GH axis consistently incorporate two characteristic features: secretion is paced by the activity of GHRH neurons, and increasing somatostatin (SST) levels delay the inter-pulse intervals of GHRH secretion^3^,^4^,^5^,^6^,^7^. GHRH neurons are not inherently rhythmic at the electrical level, since patch-clamp studies in situ failed to reveal the presence of electrical oscillations in cell bodies^8^,^9^. Furthermore, central or peripheral stimulation of the GH axis^8^,^9^, as well as hypoglycemic challenge^2^, has been shown to increase GHRH neuron spike-discharge, but with no real evidence of specific patterning. Thus, the simple scaling-up or –down the firing activity of GHRH neurons appears to be a robust mechanism involved in the control of pituitary GH secretion.

GHRH neurons also receive abundant synaptic inputs from both neuropeptide (i.e. SST)- and neurotransmitter (i.e. GABA and glutamate)-releasing neurons^8^,^10^. Moreover, GHRH neurons express the relevant receptors, including SST1 and SST2 somatostatin receptor subtypes. Hence, GHRH neurons of may in fact be capable of displaying patterned/rhythmic electrical discharges, and this may stem from differential responsiveness to their afferent SST-, GABA- and glutamate inputs. The identification of the mechanisms underlying GHRH neuron activity are important, since defects in pulsatile GH release are associated with disorders of growth and impaired responses to hypoglycemia during diabetes^1^,^11^.

In the present study, we show that SST inhibited GHRH neuron electrical activity in brain slices from GHRH-GFP transgenic mice^8^,^9^. Notably, this inhibitory effect of SST was not sustained, leading to the emergence of oscillations in GHRH neuronal population activity. Mechanistically, these firing patterns depend on a basic circuit consisting of: i) a neuropeptide input (SST); ii) a parvocellular neuronal target (GHRH neuron); and iii) neuronal inputs releasing GABA and glutamate that allow GHRH neurons to escape SST-blockade. This hitherto unidentified tripartite system may thus form coherent and incoherent feed-forward loops^12^, which recur throughout the arcuate nucleus to promote rhythms in GHRH release in response to physiological demands.

## Materials and Methods

### Study approval

Animal procedures complied with the welfare guidelines of the European Community and were ethically approved by the Direction of Veterinary departments of Herault, France (agreement number 34.251) and the Languedoc Roussillon Institutional Animal Care and Use Committee, France (#CE-LR-0818).

### Slice preparation for electrophysiological recordings

Adult 12–16 week-old GHRH-GFP mice^8^ or *sst* knockout mice^13^ were anesthetized by isoflurane inhalation, killed by decapitation, and brains quickly removed into cold (0–2 °C) solution-1 [in mM; 92 N-methyl-D-glucamine-Cl, 2.3 KCl, 1 CaCl_2_, 6 MgCl_2_, 26 NaHCO_3_, 1.2 KH_2_PO_4_, 25 glucose, 0.2 ascorbic acid, 0.2 thiourea; pH 7.4 gassed with 95% CO_2_, 5% O_2_]^8^. Sagittal sections (300 μm-thick) were cut with a microtome (Integraslice 7550, Campden Inst., UK) and stored at 34 °C in solution-2 [in mM; 115 NaCl, 2.5 KCl, 1 CaCl_2_, 4 MgCl_2_, 26 NaHCO_3_, 1.25 NaH_2_PO_4_, 25 glucose, 0.2 ascorbic acid, 0.2 thiourea; pH 7.4, gassed with 95% CO2, 5% O_2_] for at least 45 min.

### Patch-clamp recordings

Slices were immobilized with a nylon grid in a submersion chamber on the stage of an upright microscope (Axioskop FS2, Carl Zeiss) and superfused with solution-3 [in mM; 125 NaCl, 2.5 KCl, 2 CaCl_2_, 1 MgCl_2_, 26 NaHCO_3_, 1.25 NaH_2_PO_4_, 12 glucose; pH 7.4, gassed with 95% CO_2_, 5% O_2_] at a rate of 1.6 ml/min for at least 15 min at 30–32 °C. We used similar temperatures to those employed during recordings of pituitary slices, so we could match up any alterations in GHRH firing with our previous studies on GH network function^14^. Infrared differential interference contrast illumination was used to visualize neurons, with a x63 immersion objective and Nomarski differential interference contrast optics, and the images captured with an infrared camera (C2400, Hamamatsu Photonics, Massy, France). Borosilicate glass pipettes were connected to the head stage of an EPC-9/2 amplifier (HEKA, Ludwigshafen/Rhein, Germany) to acquire and store data using Pulse 8.09 software (HEKA). As indicated, agonists were either bath-applied or ejected locally. When bath-applied, solutions were changed by switching the supply of the perfusion system. The latency of the superfusion change was verified on a daily basis. When ejected in the vicinity of the neurons with a glass pipet, compounds were diluted in a HEPES-based medium containing in mM: 138 NaCl, 2.5 KCl, 2 CaCl_2_, 1 MgCl_2_, 3 NaHCO_3_, 1.25 NaH_2_PO_4_, 10 HEPES, 12 glucose, pH 7.4 with NaOH. Slices were discarded after being exposed to an agonist. All chemicals were from Sigma-Aldrich (L’isle d’Abeau, France) except D-glucose (Euromedex, France).

*For extracellular recordings* of spontaneous action potentials, pipettes (5–7 MΩ) were filled with (in mM), 130 NaCl, 2.5 KCl, 10 HEPES, 10 Glucose, 2 CaCl_2_, 1 MgCl_2_, pH 7.4 with NaOH (295 mOsm adjusted with NaCl). Neuronal activity was recorded in the voltage clamp mode (0mV) of the loose-patch configuration^8^. Standard off-line detection of spontaneous action potentials was performed with Axograph 4.0 (Axon Instruments Inc., Foster City, CA). In brief, a template was generated and used to scan the raw trace for similar waveforms. All matching events were stored and, when present, false positive events were discarded, either manually or automatically on the basis of their amplitude or kinetics. Other calculations and analysis were performed with IgorPro (Wavemetrics, Lake Oswego, OR). Mean action potential rate was calculated every 5 s, over the whole time course of the experiments, and normalized to the control level. For the quantification of GHRH neuronal population activity, the results of all similar experiments were aligned and averaged with respect to the time of the solution change. Intrinsic and inter-individual heterogeneity indices were calculated as |rate(t_n_) − rate(t_n−1_)|and |rate(t_n_) − mean rate(t_n_)|, respectively, for each data point. For the quantification of early and late effects of SST, the mean firing rates were calculated every 60 s, over the time course of the experiments. The amplitude of the early or late effects was selected as the maximal change occurring, respectively, during the first 10 min or 10–20 min following agonist application.

*For whole cell recordings*^8^, pipettes (6–8 MΩ) were filled with solution containing in mM: 2.25 KCl, 125.3 KMeSO_3_, 10 HEPES, 0.1 EGTA acid, 1 MgCl_2_, 2 MgATP, 0.5 Na-GTP, 5 Na_2_-phospocreatine, 2 Na-pyruvate, 2 malate, pH 7.2 with KOH (295 mOsm adjusted with KMeSO_3_). In the current-clamp mode, the spontaneous fluctuations of the membrane potential were measured at 0 pA. The detection and calculation of the firing rate was identical as above, using the appropriate template, except that it was averaged every 2 s. For the calculation of the resting potential, time-series were filtered at 2 Hz, eliminating all peaks, and the amplitude averaged every 2 s.

In the voltage-clamp mode, steady-state or synaptic currents were recorded. Both spontaneous GABAergic, GABAzine-sensitive and glutamatergic, 6-cyano-7-nitroquinoxaline-2,3-dione (CNQX)-sensitive synaptic currents were captured at −30 mV and −70 mV, respectively^8^. Miniature currents were recorded under the same conditions as synaptic currents, in the presence of 500 nM tetrodotoxin^8^. These events were extracted as described above for action potentials, using the appropriate template and filters, and their amplitudes, inter event intervals and densities (amplitude × instantaneous frequency) were plotted. For the calculation of mean values (amplitude, intervals, density), 60 s data bins were averaged from identical experiments with respect to the agonist application. Early and late maximal effects of SST were calculated during the first 5 min and 10–20 min after agonist application. For steady-state recordings, neurons were voltage clamped at −50mV and peak current and current at the end of SST application calculated. Decay rate was steady state current - peak current/steady state time - peak time. Similar experiments were performed in the presence of 6,7-dinitroquinoxaline-2,3-dione (DNQX) + GABAzine with identical results (data not shown).

### Statistics

Normality was tested using the D’Agostino Omnibus test (Graphpad Prism). In the bar graphs, data were expressed as mean ± SEM and compared with a paired Student’s *t* test, using the appropriate sets of values. For the kinetics analysis of firing rates, data were expressed as mean ± SEM at each time point and compared with a paired Student’s *t* test, to delineate the ranges of differences between control and agonist-treated distributions (p < 0.01 was taken as significant; *ns*, not significant). Mean distributions are represented as lines connecting the mean values, and error bars represent the SEM. For clarity, only a representative portion of the mean ± SEM values are shown in the graphs. Multifactorial comparisons of the data sets (between genders or drugs) were performed with a two-way repeated measure ANOVA (Graphpad Prism), or for non-parametric data, an ANOVA-type statistic (R Project)^15^. Comparisons between mean unpaired distributions were performed with a Mann-Whitney test (Graphpad), as indicated. In all cases, p < 0.05 was considered as significantly different.

## Results

### Acute somatostatinergic inhibition of GHRH neurons depends on GIRK activation

**Figure 1:**
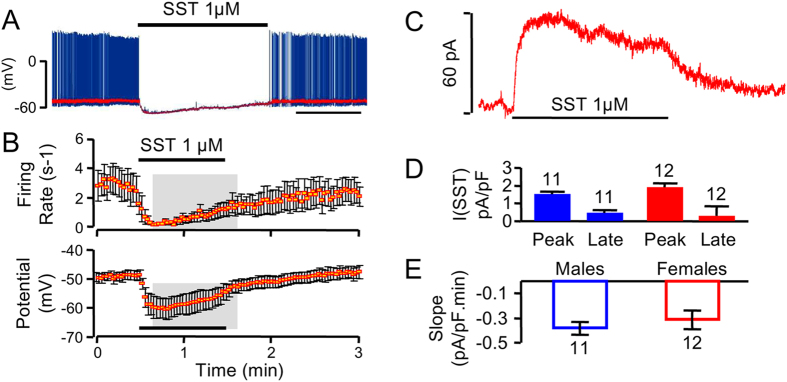
SST rapidly silences GHRH neuron activity. (**A**) Representative trace showing silencing of an identified GHRH neuron following local pressure ejection of somatostatin (SST). (**B**) Mean effects of SST treatment on spontaneous action potential firing (top) and resting potential amplitude (bottom) in GHRH neurons from male animals (*n* = 7). Data are averages of 2 sec bins. Statistically significant differences *versus* control (before SST application) are highlighted by the white area (P < 0.05, paired Student’s *t* test). (**C**) 5 min SST exposure induced an outward current in a GHRH neuron held at −50 mV. (**D**) Mean SST-induced current densities in GHRH neurons at the onset (peak) and end (late) of 1 μM SST ejections. Males are shown in blue and females in red. (**E**) As for (**D**) but mean decay rates of the SST-induced current densities.

SST has been shown to induce profound electrical silencing through activation of G protein-gated inwardly rectifying potassium (GIRK)-currents^16^. Consistent with this, focal application of SST to GFP-tagged GHRH neuron somata triggered a transient hyperpolarizing response, which inhibited action potential firing in both male and female hypothalamic slice preparations (Fig. 1A,B). Furthermore, these SST responses could be prevented by bath application of barium ions (200 μM) to block GIRK channel conductance^16^,^17^. Quantification of SST-induced K^+^-currents (Fig. 1C) revealed no differences in either current amplitude or inactivation decay rates between males and females (Fig. 1D,E), suggesting that GHRH neurons respond to acute SST application in a sex-independent manner.

### Tonic somatostatin stimulation unveils delayed GHRH neuron firing patterns

**Figure 2:**
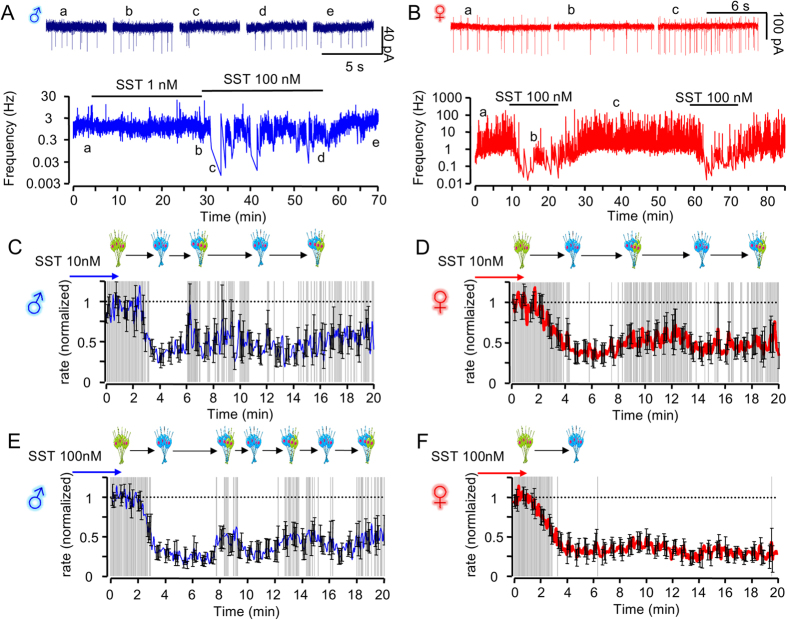
SST induces delayed oscillations in GHRH neuron spike firing. (**A**) Action potential firing frequency of a GHRH neuron from a male animal during SST superfusion (raw traces, above). (**B**) As for (**A**) but female animals. (**C**) Mean traces showing effects of 10 nM SST (applied *t* = 0 min) on spontaneous GHRH neuron action potential firing kinetics in male animals (*n* = 15). Frequencies are normalized (1 = max, 0 = min). Grey (P > 0.01) and white (P < 0.01) shaded areas indicate significant differences in GHRH population electrical activity *versus* control (hatched line) (paired Student’s *t* test). The schematic above the traces shows the nature of the pooled population responses (ON, green; OFF, blue; heterogeneous, blue/green). (**D**) As for (**C**) but female animals (*n* = 19). (**E**) As for (**C**) but responses to 100 nM SST (*n* = 14). (**F**) As for (**E**) but female animals (*n* = 13). The control action potential frequencies were 1.92 ± 0.5 Hz in (**C**); 1.61 ± 0.5 Hz in (**D**); 2.61 ± 0.6 Hz in (**E**); and 2.24 ± 0.4 Hz in (**F**).

To study longer-lasting effects of SST on neuronal activity^18^, GHRH electrical activity was monitored over dozens of minutes. Prolonged local ejection or bath application of SST both resulted in a transient hyperpolarization due to recurrent episodes of heightened GHRH neuron spiking activity (Fig. S1). These irregular patterns were unlikely to be associated with dilution of intracellular contents by the pipette solution, as identical results could be detected using loose patch-clamp to rule out cell dialysis artefacts^19^ (Fig. 2A). While the temporal profiles subtly differed from one neuron to another, the observed pattern in a given neuron was robust, since it could be reproduced by repeat application of SST (Fig. 2B). On average, SST 10 nM did not induce a tonic inhibition in the majority of recorded neurons, in both males (Fig. 2C and Fig. S2A,C) and females (Fig. 2D and Fig. S2B,D). As a result, the GHRH population was able to escape from the inhibitory effects of SST. These alterations in mean firing activity were more rapid, frequent and varied in males compared to females (Fig. 2 and Fig. S3). Of note, analysis of the SEMs over time revealed sudden increases in the heterogeneity of the neuronal responses in males (Fig. 2C,D and Fig. S3A), and this was associated with significant differences in the intrinsic heterogeneity index (Fig S3B). Even at a higher SST concentration (100nM), electrical activity was still not homogenous in GHRH neurons of males, while it was severely attenuated to about 25% of its initial level in females (Fig. 2E,F and Fig. S3C). Indeed, at this concentration, the inter-individual heterogeneity index was higher in males versus female mice (Fig. S3D).

### Sst1 and sst2 receptor activation mediates GHRH neuron firing patterns in response to somatostatin

**Figure 3:**
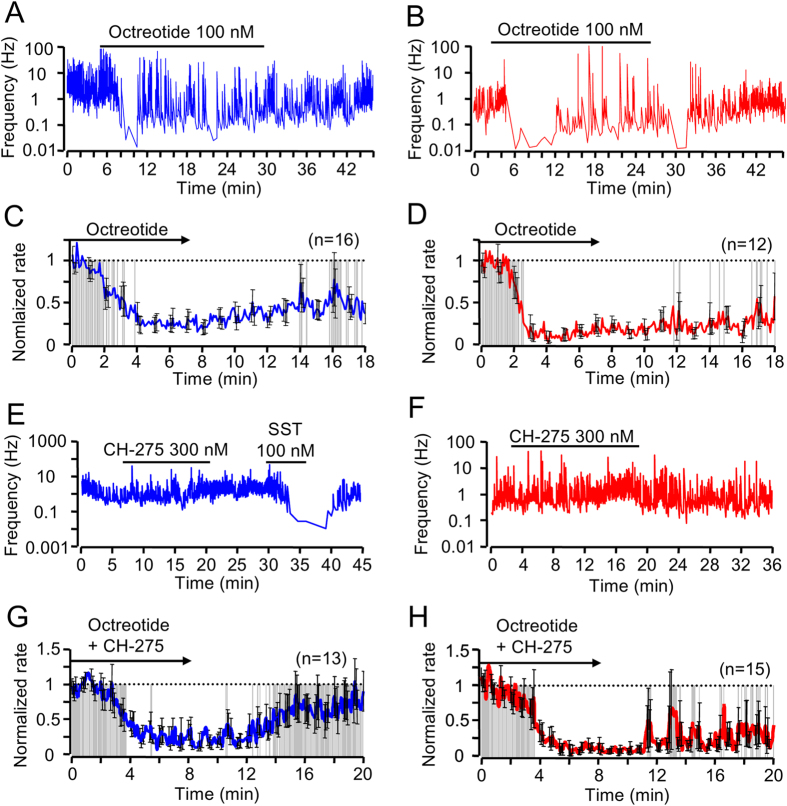
SST acts through sst1 and sst2 to inhibit GHRH neuron activity. (**A**) 100 nM Octreotide superfusion suppresses action potential firing frequency in male GHRH-GFP neurons. (**B**) As for (**A**) but female animals. (**C**) Mean traces showing GHRH neuron action potential firing kinetics in male animals following application of the sst2 agonist octreotide (*n* = 16). Frequencies are normalized (1 = max, 0 = min). Grey (P > 0.01) and white (P < 0.01) shaded areas indicate significant differences in GHRH population electrical activity *versus* control (hatched line) (paired Student’s *t* test). (**D**) As for (**C**) but female mice (*n* = 12). (**E**) Bath application of the sst1 agonist CH-275 does not alter GHRH neuron action potential firing rate in male animals. SST was used as a positive control. (**F**) As for (**E**) but female animals. (**G**) Mean traces showing delayed recovery from octreotide suppression in CH-275 treated male GHRH neurons (*n* = 13). (**H**) As for (**G**) but showing appearance of recurrent spiking activity in females (*n* = 15). In all cases, compounds were introduced at t = 0 min. The control action potential frequencies were 3.22 ± 0.8 Hz in (**C**); 1.38 ± 0.2 Hz in (**D**); 1.53 ± 0.4 Hz in (**G**); and 1.97 ± 0.4 Hz in (**H**).

GHRH neurons express sst1 and sst2 receptors, and both subtypes are implicated in the control of GH secretion^10^,^20^,^21^,^22^. To investigate the relative contribution of these receptors to SST-induced oscillations in GHRH neuron electrical activity, selective agonists were employed^23^. Application of the sst2 agonist octreotide diminished the firing rates of GHRH neurons in slices derived from both male (Fig. 3A and Fig. S2E) and female GHRH-GFP mice (Fig. 3B and Fig. S2F). Although this inhibition was not necessarily homogenous at the single neuron level (Fig. 3A,B), the blunting effect of octreotide was tonic at the population level, with only few significant escapes from sst2ergic inhibition in both male and female mice (Fig. 3C,D). Despite this, the heterogeneity of the responses was still slightly higher in males compared to females (Fig. S4A,B).

Next, we examined the effects of CH-275, an sst1 receptor agonist^23^,^24^,^25^. Application of CH-275 failed to significantly decrease spike frequency in both sexes (Fig. 3E,F and Fig. S2G,H), while SST was effective at modulating spike firing in the same experiments (Fig. 3E). However, when octreotide and CH-275 were co-applied, with the aim of mimicking the effects of SST, the mean behavior of the GHRH neurons was a prolonged and irregular inhibition in males (Fig. 3G and Fig. S2I), whereas shorter episodes of recurrent spiking were detected in females (Fig. 3H and Fig. S2J). Accordingly, firing rate heterogeneity (SEM) was enhanced by CH-275 in both males and females (Fig. S4C,D), but this effect was significant over a prolonged period of time in males only (i.e., from 8 to 20 minutes, Fig. S4C). These findings suggest that the pattern induced by SST occurs as a result of a dual activation of sst1 and sst2 receptors. Excluding a confounding role for endogenous SST release, experiments with SST, CH-275 and octreotide were repeated in sst knockout mice^3^,^13^, with similar results (Fig. S5). These results suggest that sst2 activation might account for the tonic inhibition induced by SST, while simultaneous sst1 recruitment would promote the irregular, rebound-like, pattern.

### Glutamatergic and GABAergic inputs set the tempo in GHRH neurons upon sustained somatostatin stimulation

**Figure 4:**
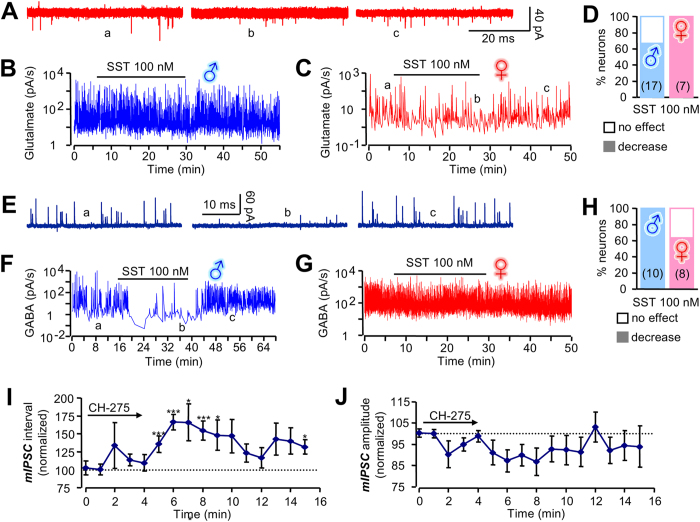
Inhibition of GHRH neuron synaptic currents by SST. (**A**) Raw traces showing spontaneous glutamatergic currents in female GHRH neurons held at −50mV (a, b and c represent before, during and after SST, respectively, and correspond to the regions shown in (**C**)). (**B**) Glutamatergic current frequency in male GHRH neurons following 100 nM SST superfusion. (**C**) As for (**B**) but showing a reduction in current density in SST-treated female slices. (**D**) Bar graph showing proportion of GHRH neurons displaying reduced glutamatergic currents in response to SST (*n* = 17 for males and *n* = 7 for females). Mean latencies for the effects were 3.4 ± 0.4 min and 4.6 ± 0.5 min in males and females, respectively. (**E**) Raw traces showing spontaneous GABAergic currents in male GHRH neurons (a, b and c represent before, during and after SST, respectively, and correspond to the region shown in (**F**)). (**F**) SST reduces GABAergic current density in male GHRH neurons. (**G**) As for (**F**) but showing a less potent action of SST to reduce currents in females. (**H**) Bar graph showing proportion of GHRH neurons displaying reduced GABAergic currents in response to SST (*n* = 10 for males and *n* = 8 for females). Mean latencies for the effects were 3.8 ± 0.6 min and 3.8 ± 0.7 min in males and females, respectively. (**I,J**) The sst1 agonist CH-275 increased miniature GABAergic current (mIPSC) intervals, but not their amplitude, in male GHRH neurons (recorded in the continued presence of 500nM tetrodotoxin). *P < 0.05 and ***P < 0.005 *versus* control (t = 0 min) (paired Student’s t- test). Under control conditions, intervals (**I**) and amplitudes (**J**) were respectively 0.4 ± 0.1 s and 16 ± 3 pA.

The spontaneous firing activity of GHRH neurons is mainly driven by a local (hypothalamic) balance between glutamatergic (excitatory) and GABAergic (inhibitory) neuronal inputs^8^,^9^. We therefore sought to determine whether SST-induced GHRH firing patterns could be associated with indirect synaptic effects. In both sexes, SST inhibited both glutamatergic (Fig. 4A–C and Fig. S6A,B) and GABAergic (Fig. 4E–G and Fig. S4C,D) synaptic currents after a lag period of a few minutes. Notably, SST robustly decreased the magnitude of glutamatergic inputs in all females, but only two thirds of males (Fig. 4D). Suggesting that these properties were sexually dimorphic, the opposite convention was observed for GABAergic currents (Fig. 4H). By contrast, when present, the inhibitory effects of SST on glutamatergic currents in GHRH neurons possessed similar properties in females (7 out of 7 neurons) and in males (10 out of 17 neurons). The inhibitory effects of SST on GABAergic transmission were also similar in the responsive neurons in females (5/8) and males (10/10). From this, the strength of SST-induced inhibition on GHRH neuronal population electrical activity would be expected to be underlined by the proportion of neurons sensitive to synaptic modulations.

Therefore, we further explored whether sst receptor activation was able to generate rhythmic electrical activity in GHRH neurons through delayed modulation of glutamatergic and GABAergic currents. While glutamatergic input almost entirely accounted for GHRH neuron excitation (Fig. 4D), an analysis of these currents failed to detect a sustained coordination at the multi-neuronal level (Fig. S7A,B). By contrast, GABAergic currents were coordinated during a long-lasting application of SST (Fig. S7C), suggesting that they may play a critical role in driving oscillatory GHRH neuron firing patterns. Moreover, the sst1 agonist CH-275 increased GABAergic miniature current (mIPSC) interval but not amplitude (Fig. 4I,J), supporting a role for presynaptic modulation of GHRH neuron GABAergic synapses. Together, these findings suggest that the time course of presynaptic modulation (*i.e.* inhibition of inhibitory inputs) was similar to that of the spike patterns detected during SST application (Fig. 2A).

## Discussion

We show here the existence of long-lasting rhythms of electrical activity in GHRH neurons exposed to SST. These irregular episodes of spike firing involved activation of both sst1 and sst2 receptors, and could only be evidenced at the population level. Strikingly, these patterns in GHRH neuron activity displayed a sexual dimorphism, and this was primarily attributable to a sex-dependent control of GABAergic and glutamatergic inputs by SST, rather than intrinsic differences in the GHRH neurons themselves. We thus hypothesize that simple tripartite hypothalamic circuits underlie growth and metabolism by pacing GHRH output.

GHRH neurons are unlikely to express the inherent capability to generate recurrent episodes of electrical activity *in situ*^8^,^9^. This is not unexpected, however, since a variety of neurotransmitters and hormones are able to modulate the electrical activity of GHRH neurons in the arcuate nucleus, and/or to modulate GHRH secretion at the median eminence^20^. So far, none of these factors, including carbachol, ghrelin, NPY, SST, and glucose have been found to orchestrate oscillations in GHRH neuronal activity^2^,^4^,^7^,^8^,^9^,^20^. The build-up of such firing patterns occurs at the population level when sustained SST stimulation activates a hypothalamic circuit comprised of GHRH neurons, together with their GABAergic and glutamatergic inputs. Various studies, including the present one, show that SST requires the activation of both sst2 and sst1 receptors^10^,^20^,^21^,^22^,^26^, and GHRH neurons express both receptor types^5^,^21^,^24^,^25^. Sst1 receptors appear tightly involved in the SST-ergic control (inhibitory) of GABAergic inputs^24^,^25^, which is likely to take place at the presynaptic level, being effective at modulating the intervals and not the amplitude of miniature GABAergic currents. Given that glutamatergic inputs are able to depolarize cell bodies beyond the threshold for action potential firing via summation of small-amplitude EPSPs^27^, such a circuit appears to be robust enough to support patterned electrical activities. In the present study, we provide pharmacological evidence for the involvement of sst1 and sst2 receptors in GHRH neuron rhythmicity, in agreement with prior anatomical, biochemical and physiological studies showing their role in the central regulation of GH secretion. While octreotide binds equally to sst2 and sst5 receptors, we think the latter subtype is unlikely to contribute to the present observations, since expression is largely limited to the cerebellum and pituitary^28^.

### Revisiting how hypothalamic GHRH neurons deliver a spike firing code

**Figure 5:**
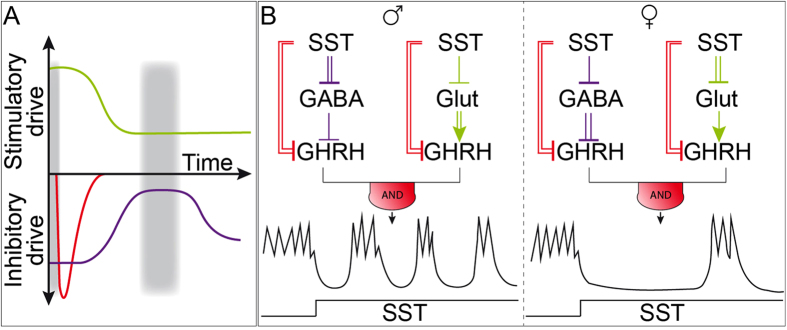
Schematic of the feedforward loops proposed to underlie GHRH rhythm generation by SST. (**A**) The feedforward loop (FFL) allows precise temporal control over GHRH neuron electrical activity via dynamic effects on inhibitory and stimulatory drive. Strengths of GIRK activation, glutamatergic and GABAergic inputs are colored in red, green and violet, respectively. Grey shaded areas illustrate episodes of GHRH neuron firing. (**B**) At the circuit level, the FFL motifs consist of a primary regulator, SST, which inhibits secondary regulators (glutamatergic or GABAergic inputs) that synapse with GHRH neurons. The network motif involving glutamatergic inputs provides a delayed excitatory source (coherent FFL; see^12^ for definitions), whereas the motif with GABAergic inputs acts as a pulser (incoherent FFL). Both sst1 and sst2 receptors are involved in SST responses, with sst1 receptors notably acting to intermittently delay GABA current onset (pulse generator). The sexually dimorphic spiking rhythms recorded in GHRH neurons were associated with a sex-dependent SST-regulation of linked FFLs but not GIRK currents in GHRH neurons (signs colored in red). Signs for inhibitory and stimulatory interneuron effects are colored in violet and green, respectively. Sign thickness represents the response magnitude. The “AND” gate sign represents the link between both coherent and incoherent FFLs, which recurs within the arcuate nucleus.

Figure 5A schematizes how SST inhibition might allow glutamatergic and GABAergic inputs to form a tripartite hypothalamic circuit with GHRH neurons. This likely involves the following events:

- *Step 1: Early and transient hyperpolarization of GHRH neurons*. Altogether, our results suggest that sst2 receptors recruit GIRK channels with a short latency, switching off GHRH neuron action potential firing. GIRK channels, which inactivate/desensitize within a few mins, likely participate in an early transient inhibition of GHRH neurons.
- *Step 2: Delayed and sustained inhibition of GHRH neuron activation.* Tonic SST receptor activation leads to a delayed and long-lasting decrease in glutamatergic transmission.
- *Step 3: Delayed and temporary dis-inhibition of GHRH neurons.* Sst receptor activation exerts a delayed suppression of the inhibitory GABA currents, as reported in other brain regions^29^. The rapid GIRK current inactivation/desensitization, together with this coordinated and temporary drop in inhibitory inputs, may be sufficient to enable spike escapes in GHRH neuron firing (Fig. 5A).

Although further studies will be needed to elucidate the precise mechanisms underlying delayed SST effects, the sequential modulation of hypothalamic interneurons may represent an initial step in the generation of patterned GHRH spike firing^6^,^30^. This modulation likely participates in the sexual dimorphism of the GH axis, as there were qualitative and quantitative differences in the SST inhibition of GHRH neurons in males and females. Briefly, it was more regular and more tonic in GHRH neurons of females than males, especially at the highest SST concentration of 100nM. At the cellular level, glutamatergic neurotransmission was an obligatory target of SST in females, providing a mechanism for a more tonic inhibition, unlike in males where it was absent in one-third of GHRH neurons. Strikingly, the SST-ergic modulation of GABA inputs was a mirror image of that in glutamatergic neurons, being especially robust and synchronized in males. Whether the proportion of synapses engaged in the modulatory effects of SST varies with time warrants further studies. This would be a flexible mechanism for adapting the central control of the GH axis during the oestrous cycle, for instance, or in response to feeding status^31^,^32^. While female mice were randomly cycling, we think it unlikely that SST-ergic-regulation of GHRH neuron firing closely follows the oestrous cycle, since the SST-ergic effect could be qualitatively different in GHRH neurons from a given mouse. Moreover, responses in male animals were more varied than those in female animals, suggesting that hormonal status may homogenize GHRH population activity. Nonetheless, further experiments, for example in ovariectomized animals supplemented with or without E2, would be required to investigate this.

### SST engages both coherent and incoherent feed-forward loops to drive rhythmic firing in GHRH neurons

The regulatory circuits reported here share features consistent with the ‘parallel inhibition’ building block model of synaptic connectivity^33^, as well as feedforward loop motifs (so-called FFL). These phylogenetically-conserved mechanisms are reported to play central roles in metabolic, transcriptional and neuronal networks^12^,^34^,^35^,^36^,^37^,^38^,^39^,^40^. Hypothalamic circuits involving stimulatory (glutamatergic) inputs may act as a coherent FFL, providing delayed responses to a persistent stimulus. Those characterized by inhibitory (GABAergic) inputs may function as an incoherent FFL, acting to pulse activity, as illustrated by the transient inhibitory effects of the sst1 agonist at the synaptic GABAergic level (see Fig. 4I). Both coherent and incoherent feedforward neuronal circuits may be widespread amongst GHRH neurons (Fig. 5B), although they are not a prerequisite. Indeed, in response to SST, a proportion of GHRH neurons did not exhibit glutamatergic or GABAergic modulations in males and females, respectively. Nevertheless, SST is essential for the sexual dimorphism of GH secretion, suggesting that a similar circuitry may operate *in vivo* to mediate growth and metabolism^3^.

## Conclusion

The present study unveils local hypothalamic connections that form FFLs to determine rhythmic firing patterns in GHRH neurons. Such discrete hypothalamic circuits may be important sites of sex-imprinting of GH axis output, leading to divergent metabolic traits in males and females^20^,^41^,^42^,^43^,^44^, as well as representing a target for the central mechanisms underlying GHRH-dependent regulation of glucose homeostasis and food intake^2^,^45^,^46^. Consequently, our results shift the focus from the GHRH neurons themselves, towards the glutamate and GABA neurons that drive GHRH secretion.

Further studies are now warranted to explore whether interplay between FFLs may be important for coding information in other sets of hypothalamic neurons that receive glutamatergic and GABAergic inputs^47^,^48^,^49^,^50^ and display neuromodulation in response to neuropeptides^51^; for example, anorexigenic POMC and orexigenic AgRP neurons^52^. In addition to other regulatory mechanisms, such as tonic activity changes^2^, increased number of active neurons^53^, and modulation at the nerve terminal level^54^, FFL motifs may thus provide a mechanism^55^,^56^ to maintain hypothalamic circuit robustness in the face of perturbed metabolic homeostasis^57^,^58^.

## Additional Information

**How to cite this article**: Osterstock, G. *et al.* Somatostatin triggers rhythmic electrical firing in hypothalamic GHRH neurons. *Sci. Rep.* **6**, 24394; doi: 10.1038/srep24394 (2016).

## Supplementary Material

Supplementary Information
