# Supplementary material for: Somatostatin triggers rhythmic electrical firing in hypothalamic GHRH neurons
Source: Sci Rep. 2016 Apr 13;6:24394. doi: 10.1038/srep24394 (PMC4829871; doi:10.1038/srep24394)
Supplement: Supplementary Information [file srep24394-s1.pdf]

SUPPLEMENTARY INFORMATION

Somatostatin triggers rhythmic electrical firing in hypothalamic GHRH neurons

Guillaume Osterstock [1,2,3], Violeta Mitutsova [1,2,3], Alexander Barre [1,2,3], Manon Granier [1,2,3], Pierre Fontanaud [1,2,3], Marine Chazalon [1,2,3], Danielle Carmignac [4], Iain CAF Robinson [4], Malcolm J Low [5], Nikolaus Plesnila [6], David J Hodson [7], Patrice Mollard [1,2,3*], Pierre-François Méry [1,2,3 *]

Addresses: [1]Inserm U-1191, Montpellier, France; [2]CNRS UMR 5203, Institut de Génomique Fonctionnelle, Montpellier, France; [3]Université Montpellier, Montpellier, France; [4]Division of Molecular Neuroendocrinology, MRC National Institute for Medical Research, The Ridgeway, Mill Hill, London, UK ; [5]Department of Molecular and Integrative Physiology, University of Michigan Medical School, Ann Arbor, MI, USA; [6] Royal College of Surgeons in Ireland, Dublin 2, Ireland; [7]Section of Cell Biology and Functional Genomics, Department of Medicine, Imperial College London, Imperial Centre for Translational and Experimental Medicine, Hammersmith Hospital, Du Cane Road, London W12 0NN, United Kingdom.

* Corresponding authors: patrice.mollard@igf.cnrs.fr or pierre-francois.mery@igf.cnrs.fr

Running Head. SST-triggered neuronal FFL motifs

Supplementary Figure 1: Resumption of action potential firing in GHRH neurons during prolonged SST exposure. (**A-C**) Representative current clamp recordings of electrical activity in GHRH neurons. SST (0.1-1 µM) was either ejected via a glass pipette (**A**) or perfused into the cuve (**B,C**). Note that, in all cases, neuron electrical activity resumed after 2-4 minutes.

Supplementary Figure 2: **Inhibitory effects of sstR agonists on GHRH neuron spontaneous firing rates**. Summary of the results collected in males (left column) and females (right column). (**A-D**) Bar graphs depicting maximal effects of SST (A,B, 10 nM; C,D, 100 nM) on the mean rate of spontaneous action potentials in GHRH neurons. (**E-H**) As for (**A-D**) except in response to the sst2 agonist, octreotide (**E,F**, 100 nM) and the sst1 agonist CH-275 (**G,U**, 300 nM), respectively. (**I,J**) Bar graphs showing the effects of both octreotide (100 nM) and CH-275 (300 nM). In each case, experiment numbers are indicated. Bar and lines represent mean ± SEM of before (control = 0-5 min), during (early = 5-15 min; late = 15-25 min) and after (wash) drug application. **P<0.01 and ***P<0.005 *versus* control (paired Student's t-test). $^{\$\$}$P<0.01 and $^{\$\$\$}$P<0.005 *versus* late (paired Student's t-test).

Supplementary Figure 3: Heterogeneity of the somatostatinergic inhibition of GHRH neurons in GHRH-GFP mice. (**A,C**) Time-course of the dispersion of the spontaneous firing rates (SEM) in the presence of SST 10 nM (**A**), 100 nM (**C**) in males and females. Sex-differences: *, P<0.05, ****, P<0.0001, and $$$$, P<0.0001, differences between concentrations (Mann-Whitney test). (**B**) intrinsic- and (**D**) inter-individual heterogeneity index of the spontaneous firing rate of GHRH neurons in the presence of either SST 10 nM (**B**) or SST 100 nM (**D**) in males and females. Sex-differences: *, P<0.05 (two-way repeated measure ANOVA).

Supplementary Figure 4: Heterogeneity of the effects of SST-ergic agonist on GHRH neurons in GHRH-GFP mice. (**A**) Time-course of the dispersion of the spontaneous firing rates (SEM) in the presence of octreotide 100 nM in males and females. Sex-differences: *, P<0.05, (Mann-Whitney test). (**B**) inter-individual heterogeneity index of the spontaneous firing rate of GHRH neurons in the presence of octreotide SST 100 nM in males and females. Sex-differences: ****, P<0.001 (wwo-way repeated measure ANOVA). (**C,D**) CH-275 (300 nM) increased the dispersion of the spontaneous firing rates (SEM) in the presence of octreotide 100 nM in males and females, but this effect was significant in males only. $, P<0.05, difference between treatments in the 8-20 min range (Mann-Whitney test).

Supplementary Figure 5: Properties of the somatostatinergic inhibition of GHRH neurons in somatostatin (*sst*) knockout mice. (**A-C**) Bar graphs showing maximal effects of SST (**A**, 1 nM; **B**, 10 nM; **C**, 100 nM) on the mean rate of spontaneous action potential firing in GHRH neurons. (**D**) Representative loose patch-clamp recording of GHRH neuron spontaneous firing rates in the absence

and presence of 300 nM CH-275 (sst1 agonist). (**E**) As for (**D**) but in the absence and presence of 100 nM octreotide (sst2 agonist). (**F**) Bar graph showing maximal effects of 300 nM CH-275. (**G**) As for (**F**) but in response to 100 nM octreotide. In each case, experiment numbers are indicated. Bars and lines represent mean ± SEM of before (control = 0-5 min), during (early = 5-15 min; late = 15-25 min) and after (wash) drug application. *P<0.05 and ** P<0.01 *versus* control (paired Student's t-test). All experiments were performed in sagittal brain slices from male GHRH-GFP x *sst* -/- mice.

Supplementary Figure 6: Inhibitory effects of SST on synaptic currents in GHRH neurons. (**A**) Bar graphs summarizing inhibitory effects of SST on glutamatergic current density in male GHRH neurons. (**B**) As for (**A**) but female animals. (**C,D**) As for (**A,B**) except analysis of spontaneous GABAergic currents. In each case, experiment numbers are indicated. Densities were averaged every 60 s for each experiment. Bar and lines represent mean ± SEM of before (control = 0-5 min), during (early = 5th min; late = 15-20 min) and after (wash) drug application. *P<0.05, **P<0.01 and ***P<0.005 *versus* control (paired Student's t-test).

Supplementary Figure 7: Kinetics of the inhibitory effects of SST on synaptic currents. (**A**) SST does not significantly alter glutamatergic current kinetics (normalized density) in male GHRH neurons. (**B**) As for (**A**) but showing suppression of glutamatergic current density following application of SST to female slices. (**C**) SST suppresses GABAergic current density in male GHRH neurons. (**D**) As for (**C**) but showing no effect of SST. Under control conditions current densities were 134 ± 42 $pA.s^{-1}$ in **A**; 151 ± 42 $pA.s^{-1}$ in **B**; 117 ± 30 $pA.s^{-1}$ in **C** and 68 ± 8 $pA.s^{-1}$ in **D**. *P<0.05, #P<0.01 and $ P<0.005 *versus* control (t = 0 min) (paired Student's *t*-test).

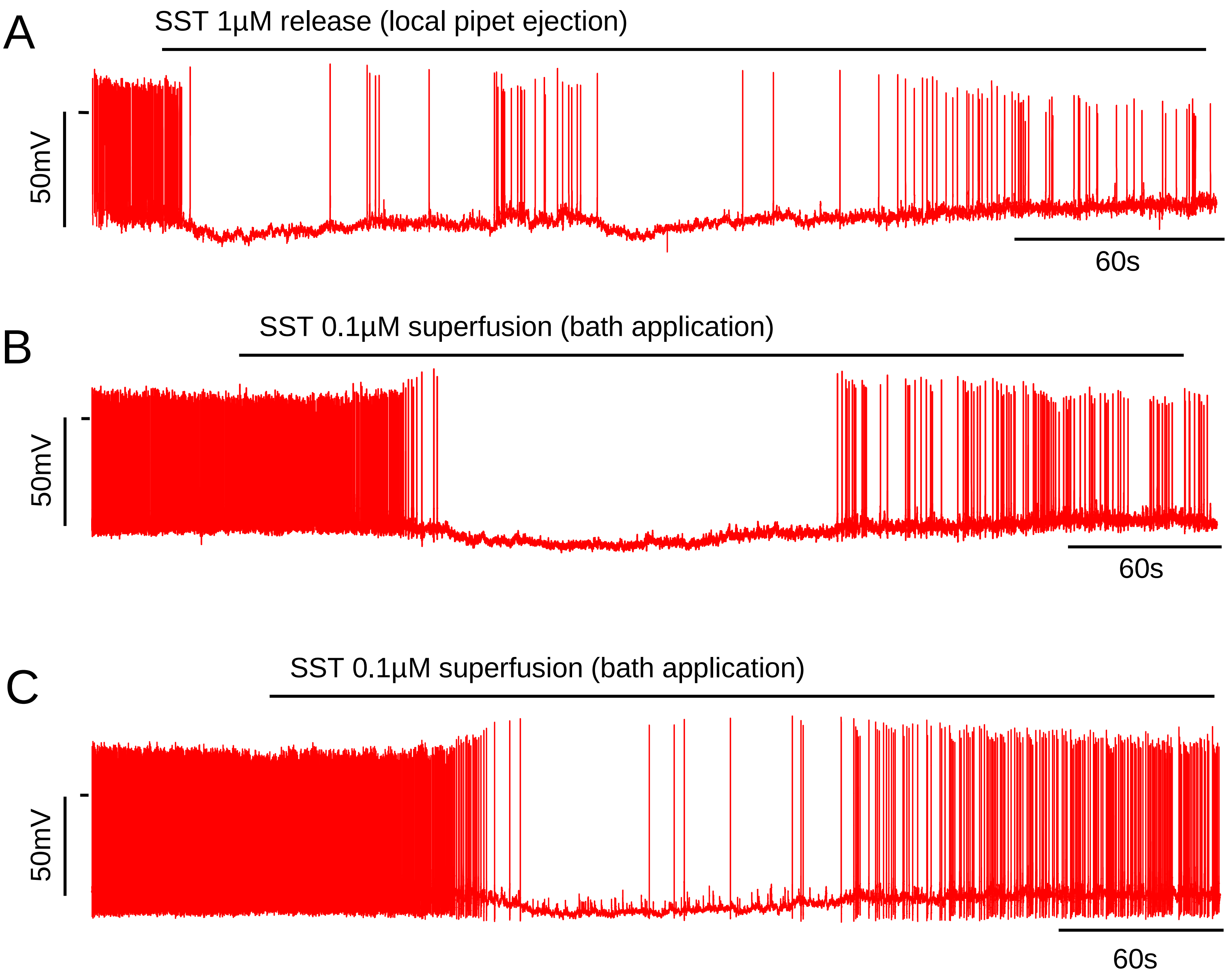
A
SST 1µM release (local pipet ejection)
50mV
60s
B
SST 0.1µM superfusion (bath application)
50mV
60s
C
SST 0.1µM superfusion (bath application)
50mV
60s

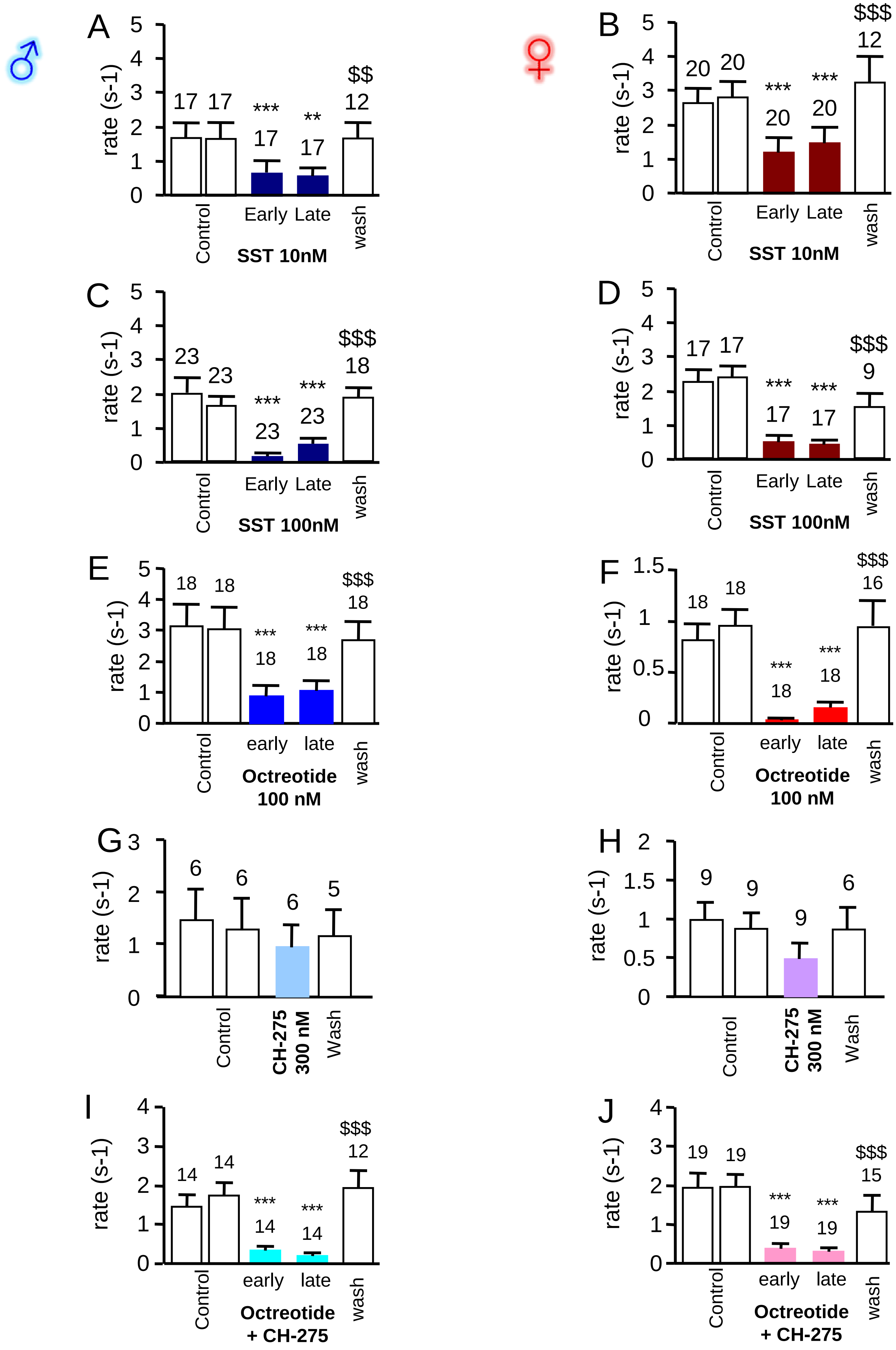
♂
♀
A
rate (s-1)
17 17
***
17
**
17
$$
12
Control
Early Late
wash
SST 10nM
B
rate (s-1)
20 20
***
20
***
20
$$$
12
Control
Early Late
wash
SST 10nM
C
rate (s-1)
23
23
***
23
***
23
$$$
18
Control
Early Late
wash
SST 100nM
D
rate (s-1)
17 17
***
17
***
17
$$$
9
Control
Early Late
wash
SST 100nM
E
rate (s-1)
18 18
***
18
***
18
$$$
18
Control
early late
wash
Octreotide
100 nM
F
rate (s-1)
18
18
***
18
***
18
$$$
16
Control
early late
wash
Octreotide
100 nM
G
rate (s-1)
6 6 6 5
Control
CH-275
300 nM
Wash
H
rate (s-1)
9 9 9 6
Control
CH-275
300 nM
Wash
I
rate (s-1)
14 14
***
14
***
14
$$$
12
Control
early late
wash
Octreotide
+ CH-275
J
rate (s-1)
19 19
***
19
***
19
$$$
15
Control
early late
wash
Octreotide
+ CH-275

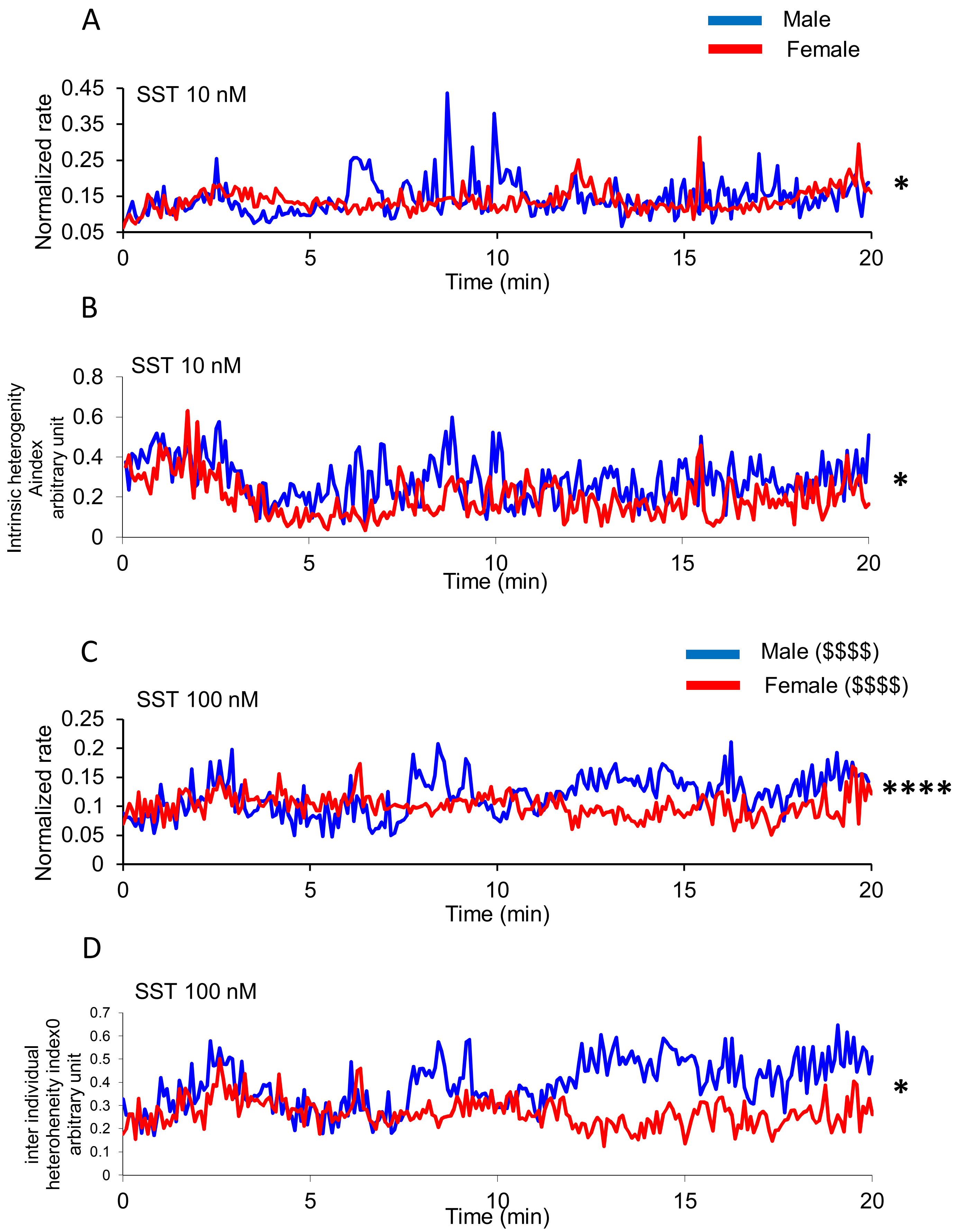
A
Male
Female
SST 10 nM
Normalized rate
0.45
0.35
0.25
0.15
0.05
0
5
10
15
20
Time (min)
*
B
SST 10 nM
Intrinsic heterogenity
Aindex
arbitrary unit
0.8
0.6
0.4
0.2
0
0
5
10
15
20
Time (min)
*
C
Male ($$$$)
Female ($$$$)
SST 100 nM
Normalized rate
0.25
0.2
0.15
0.1
0.05
0
0
5
10
15
20
Time (min)
****
D
SST 100 nM
inter individual
heteroheneity index0
arbitrary unit
0.7
0.6
0.5
0.4
0.3
0.2
0.1
0
0
5
10
15
20
Time (min)
*

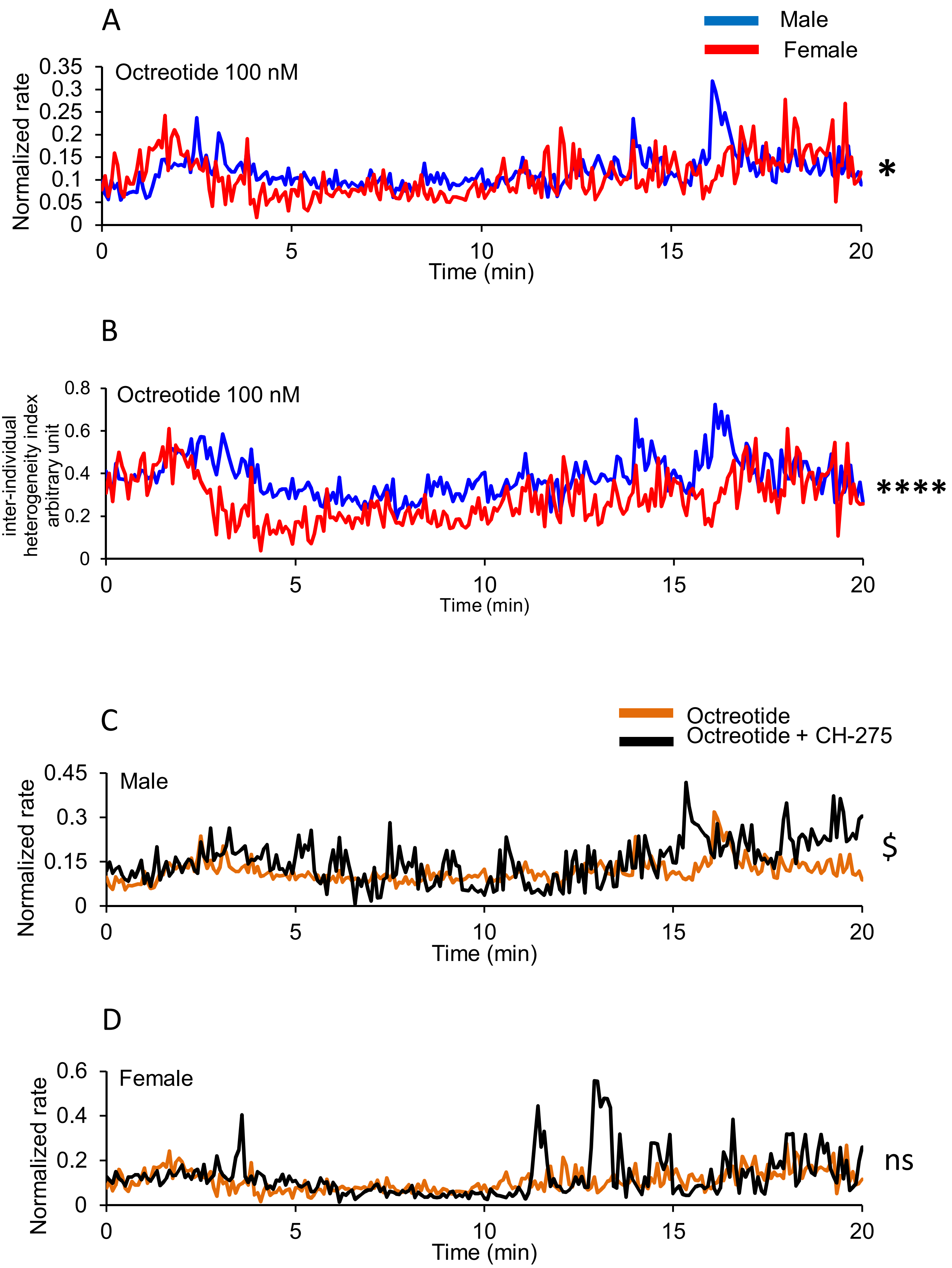

A
Male
Female
Octreotide 100 nM
Normalized rate
0.35
0.3
0.25
0.2
0.15
0.1
0.05
0
0
5
10
15
20
Time (min)
*
B
Octreotide 100 nM
inter-individual heterogeneity index arbitrary unit
0.8
0.6
0.4
0.2
0
0
5
10
15
20
Time (min)
****
C
Octreotide
Octreotide + CH-275
Male
Normalized rate
0.45
0.3
0.15
0
0
5
10
15
20
Time (min)
$
D
Female
Normalized rate
0.6
0.4
0.2
0
0
5
10
15
20
Time (min)
ns

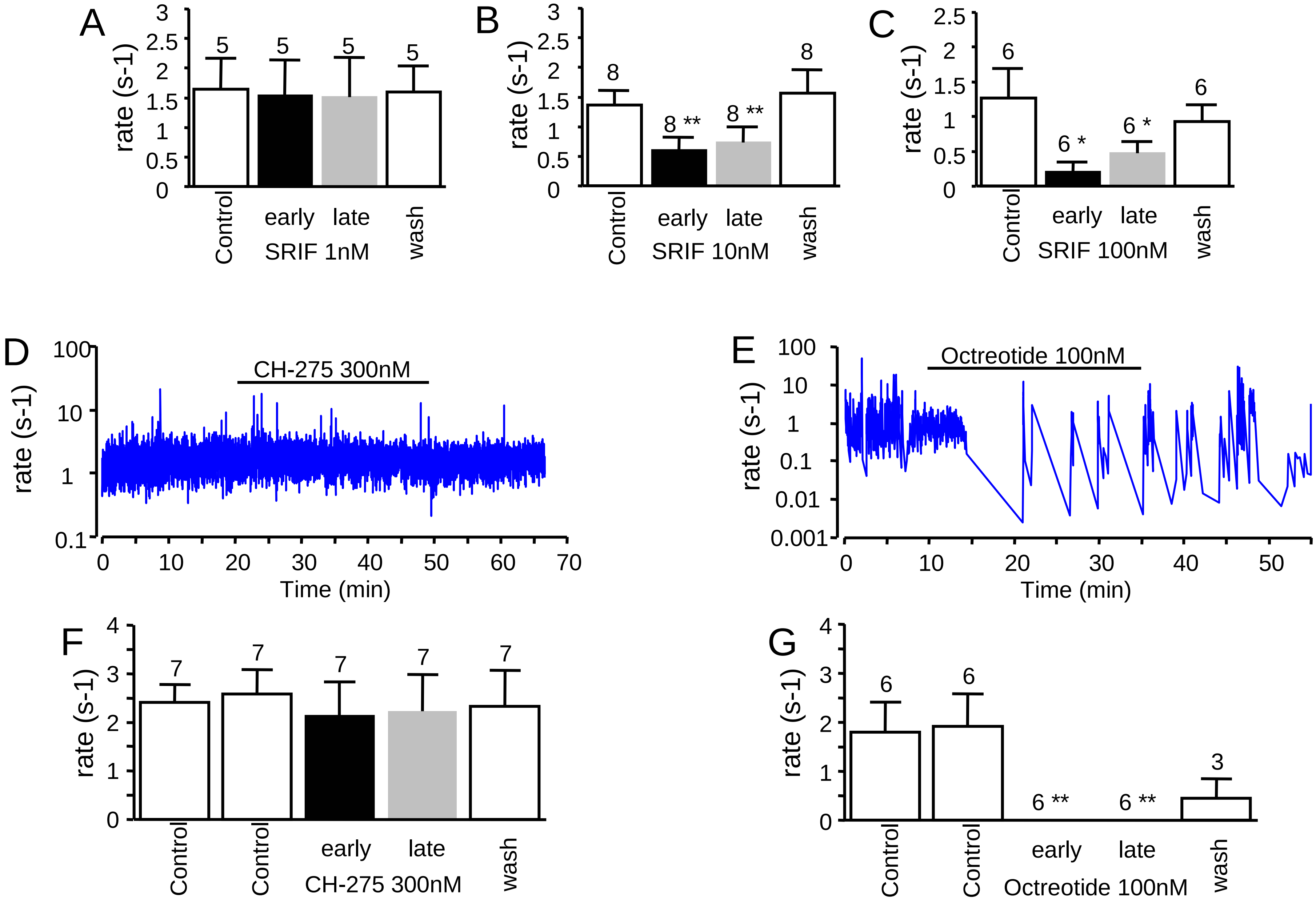
A
B
C
D
E
F
G
rate (s-1)
SRIF 1nM
SRIF 10nM
SRIF 100nM
Control
early
late
wash
8 **
6 *
CH-275 300nM
Octreotide 100nM
Time (min)
6 **

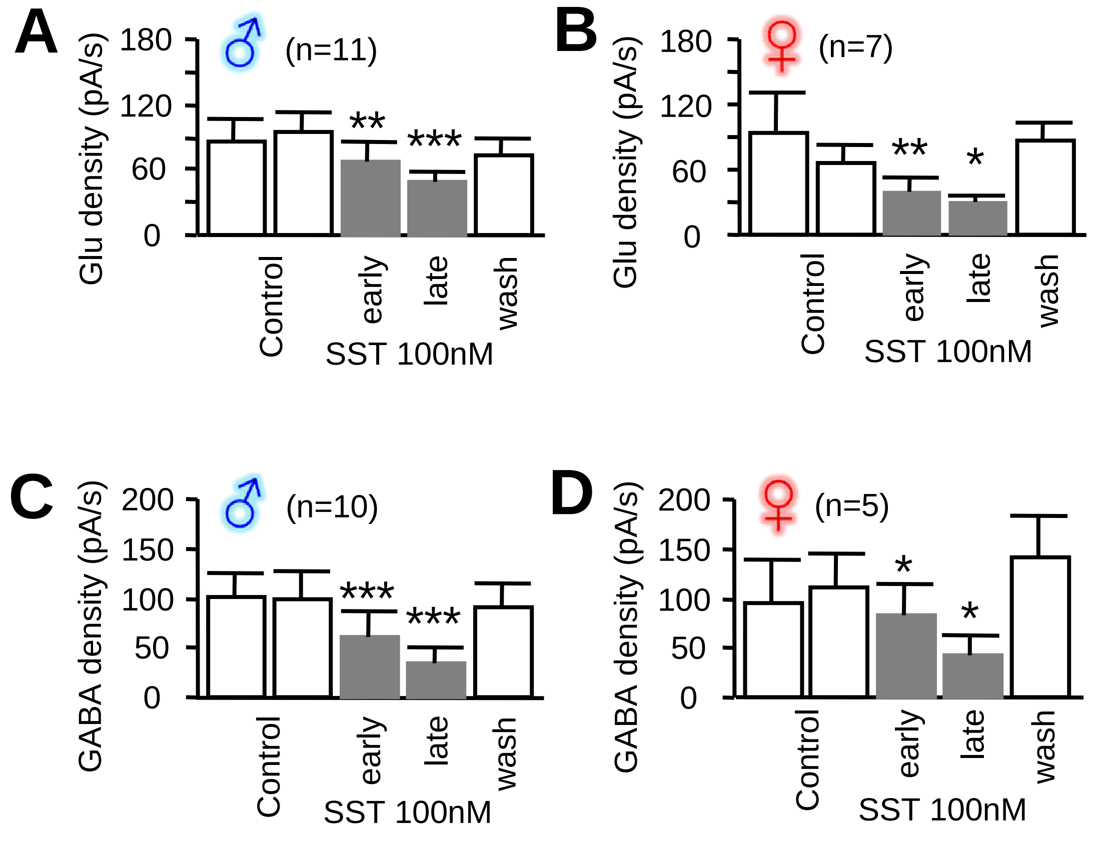
A
Glu density (pA/s)
180
120
60
0
♂
(n=11)
**
***
Control
early
late
wash
SST 100nM
B
Glu density (pA/s)
180
120
60
0
♀
(n=7)
**
*
Control
early
late
wash
SST 100nM
C
GABA density (pA/s)
200
150
100
50
0
♂
(n=10)
***
***
Control
early
late
wash
SST 100nM
D
GABA density (pA/s)
200
150
100
50
0
♀
(n=5)
*
*
Control
early
late
wash
SST 100nM

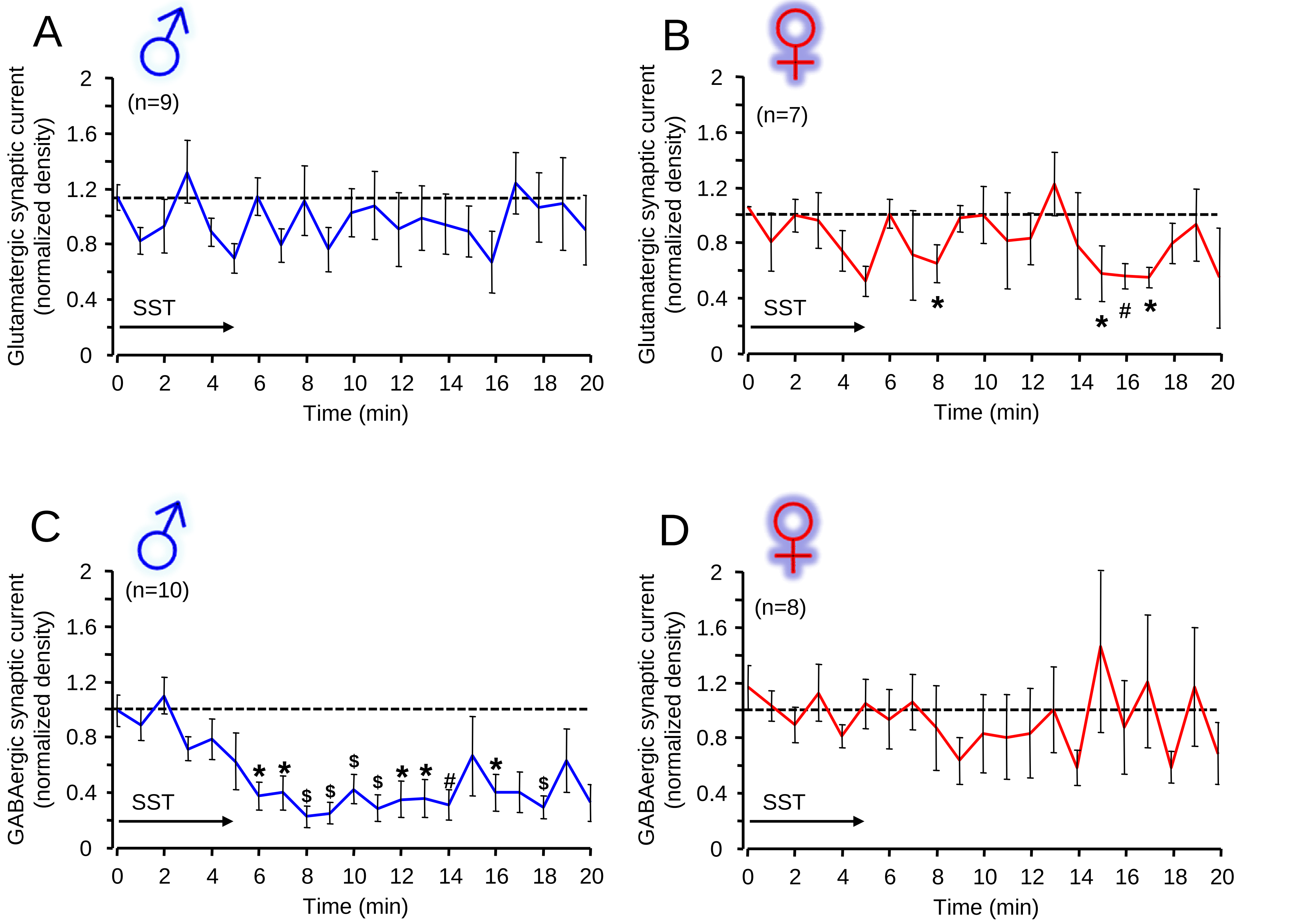

A
♂
Glutamatergic synaptic current (normalized density)
(n=9)
SST
Time (min)
B
♀
Glutamatergic synaptic current (normalized density)
(n=7)
SST
Time (min)
C
♂
GABAergic synaptic current (normalized density)
(n=10)
SST
Time (min)
D
♀
GABAergic synaptic current (normalized density)
(n=8)
SST
Time (min)
